# Langasite (LGS) Surface Acoustic Wave (SAW) Pressure Sensor with Kovar Alloy Point-Force Packaging for High-Temperature Environments

**DOI:** 10.3390/s26020567

**Published:** 2026-01-14

**Authors:** Yabing Ke, Ruoyu Zhang, Chen Fu, Jingting Luo, Zhengxi He, Zhiguang Deng

**Affiliations:** 1 Shenzhen Key Laboratory of Advanced Thin Films and Applications, Guangdong Provincial Engineering Technology Research Center for Breath Test, College of Physics and Optoelectronic Engineering, Shenzhen University, Shenzhen 518060, China; keyabing@szu.edu.cn (Y.K.); 2510232212@mails.szu.edu.cn (R.Z.); chenfu@szu.edu.cn (C.F.); 2 Shenzhen Key Laboratory of Nuclear and Radiation Safety, Shenzhen University, Shenzhen 518060, China; 3 State Key Laboratory of Advanced Nuclear Energy Technology, Nuclear Power Institute of China, Chengdu 610213, China; drsi-d40302@npic.ac.cn (Z.H.); drsi-d403-dzg@npic.ac.cn (Z.D.)

**Keywords:** langasite, SAW pressure sensor, high temperature, point-force packaging

## Abstract

Langasite (LGS)-based surface acoustic wave (SAW) sensors are promising for high-temperature pressure detection. However, their performance is limited by the low pressure sensitivity of conventional sealed-cavity packaging and temperature-induced measurement drift. To address these issues, this study introduces a novel LGS SAW pressure sensor featuring two key innovations: a Kovar alloy point-force packaging structure to amplify pressure-induced LGS substrate deformation, enhancing sensitivity compared to traditional designs, and SAW resonators fabricated on an LGS (0°, 138.5°, 26.7°) cut, selected based on electromechanical simulations for its superior intrinsic pressure sensitivity and monotonic frequency–temperature response, effectively mitigating temperature interference on pressure measurements. Experimental characterizations show the resonator achieves a high Q-value of ~3000 at ~357 MHz. Tested under conditions of 250 °C and 0–0.4 MPa, the sensor exhibits a pressure sensitivity of 0.1866 MHz/MPa with a relative error of only 4.8% versus the finite element method (FEM)-simulated 0.196 MHz/MPa, demonstrating the proposed design’s effectiveness for accurate, stable pressure monitoring in harsh high-temperature environments such as turbine engines and high-temperature manufacturing lines.

## 1. Introduction

**Figure 1:**
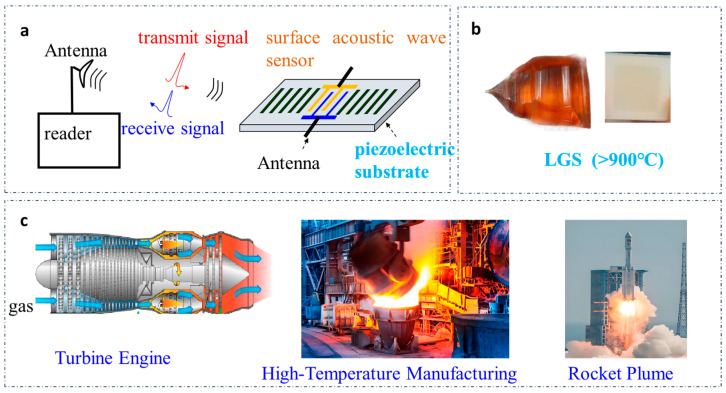
(**a**) Core working principle of SAW sensors; (**b**) LGS piezoelectric substrate; (**c**) typical high-temperature industrial conditions.

Surface acoustic wave (SAW) sensors have gained significant attention for their harsh-environment applications due to their passive operation, wireless interrogation capability, and resistance to electromagnetic interference [1,2]. As illustrated in Figure 1a, the core working principle of SAW sensors relies on antenna–reader signal transmission—where acoustic waves propagate on the piezoelectric substrate surface, and external stimuli (e.g., pressure, temperature) modulate the resonant frequency for detection—making them ideal for non-contact monitoring in extreme conditions. Among these applications, high-temperature pressure monitoring is critical for industrial scenarios such as turbine engine combustion chambers (500 °C/2 MPa) and rocket plume testing (>1000 °C), as shown in Figure 1c; however, conventional SAW substrates (e.g., quartz, LiTaO_3_, and LiNbO_3_) undergo phase transitions or decomposition above 573 °C, rendering them unsuitable for such high-temperature environments [3,4,5,6,7].

In contrast, langasite (LGS) stands out as a superior piezoelectric substrate for high-temperature SAW sensors. As depicted in Figure 1b, LGS exhibits exceptional thermal stability (withstanding temperatures up to 1470 °C) and favorable piezoelectric properties—key advantages that enable its deployment in harsh conditions where traditional substrates fail [3,4,5,6,7,8,9]. This thermal resilience has made LGS a primary candidate for addressing the unmet need of high-temperature pressure monitoring, yet existing LGS SAW pressure sensors face two unresolved limitations that hinder practical application.

First, traditional sealed-cavity packaging designs, such as those reported by Moulzolf et al. [10,11], provide insufficient pressure sensitivities for high-precision industrial monitoring (e.g., turbine engine pressure control requiring ±0.01 MPa resolution), while a point-force pressure structure that amplifies substrate deformation to boost sensitivity has been validated for quartz SAW sensors [12]. This design has rarely been adapted for LGS substrates, leaving a critical opportunity to improve sensitivity for LGS-based devices. We have previously applied this package design to enhance pressure sensitivity based on a Z-cut LGS substrate [13], but this cut is not optimal for the LGS SAW pressure sensor, indicating further work is needed. Second, temperature-induced frequency drift introduces cross-sensitivity, degrading pressure-measurement accuracy under elevated temperatures. Although the (0°, 138.5°, 26.7°) LGS cut has been identified through simulations as optimal for monotonic frequency–temperature responses [14,15,16], its integration with sensitivity-enhancing packaging to mitigate cross-sensitivity remains underexplored.

To address these limitations, this study presents a novel LGS SAW pressure sensor with two targeted innovations. First, we design a Kovar alloy packaging structure with integrated point-force application—a configuration that amplifies pressure-induced mechanical deformation of the LGS substrate, directly overcoming the sensitivity bottleneck of sealed-cavity designs. Second, we fabricate SAW resonators on the (0°, 138.5°, 26.7°) LGS cut, leveraging its superior intrinsic pressure sensitivity and stable frequency–temperature response to minimize temperature interference without additional compensation circuits.

This work validates the sensor’s performance across a temperature range of 160–400 °C (covering typical high-temperature industrial conditions shown in Figure 1c) and a pressure range of 0–0.4 MPa. The fabricated resonator achieves a high quality factor (Q-value) of ~3000 at a center frequency of ~357 MHz, ensuring low energy loss and reliable operation. Experimental results demonstrate a pressure sensitivity of 0.1866 MHz/MPa—outperforming traditional LGS SAW sensors—and a relative error of only 4.8% compared to finite element method (FEM) simulations. By resolving key limitations of existing LGS SAW pressure sensors, this study provides a robust technical solution for accurate pressure monitoring in harsh high-temperature environments like those depicted in Figure 1c.

## 2. Theoretical Analysis

### 2.1. LGS Substrate Cut and Sensitivity Simulation

**Figure 2:**
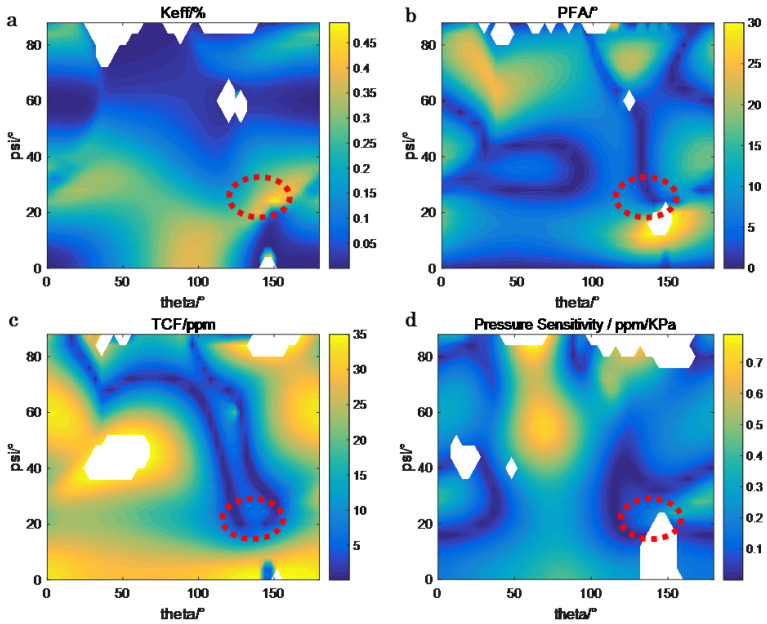
(**a**) Electromechanical coupling coefficient (Keff); (**b**) power flow angle (PFA); (**c**) temperature coefficient (TCF); (**d**) pressure sensitivity. The red circle indicates the selected cut and the white areas indicates the values that were miscalculated and thus excluded from the presentation.

For LGS-based SAW pressure sensors targeting high-temperature environments, the substrate cut must balance high pressure sensitivity with a monotonic frequency–temperature response. To identify the optimal cut, we conducted electromechanical parameter simulations using validated LGS material constants from Ref. [17], including density, stiffness, piezoelectric, and dielectric constant—values consistent with prior high-temperature SAW sensor studies [17,18,19,20,21]. The oular angle was used to define the substrate cut, where theta (θ) is the cut angle and psi (ψ) is the propagation direction. These simulations focused on four key parameters that directly influence sensor performance: the electromechanical coupling coefficient (Keff), the power flow angle (PFA), the temperature coefficient (TCF), and the pressure sensitivity. The first three parameters were calculated using the effective permittivity method, while the last parameter was calculated via the perturbation theory. These four key parameters for different cuts (θ, ψ) are visualized in Figure 2. Due to the symmetry of LGS substrate, only the ranges of θ (0–180°) and ψ (0–90°) need to be simulated.

First, Keff determines the efficiency of SAW excitation, where values exceeding 0.04 are required for strong, detectable signal amplitudes [22]. We use the formula for analyzing SAW devices: Keff = 2 × (fr − fa)/fr, where fr, fa are the resonant and anti-resonant frequencies, respectively. The (0°, 138.5°, 26.7°) cut exhibited a Keff of approximately 0.05 (Figure 2a), outperforming alternative cuts such as the (0°, 90°, 0°) cut (with Keff approximately at 0.02) and ensuring robust acoustic wave generation. Second, PFA affects the stability of the SAW propagation direction; near-zero PFA (<1°) avoids wavefront distortion that could degrade frequency accuracy [22]. The (0°, 138.5°, 26.7°) cut showed a PFA of ~0.5° (Figure 2b), enabling uniform acoustic wave propagation across the substrate surface. Third, TCF characterizes frequency drift with temperature, and a monotonic, low-variation TCF (−10 to 10 ppm/°C) is essential for minimizing cross-sensitivity. This cut exhibited a TCF of ~5 ppm/°C (Figure 2c), maintaining stable frequency responses across the target high-temperature range (160–400 °C). Finally, pressure sensitivity should be high (>0.1 ppm/KPa); when calculated via perturbation theory [23,24], which links substrate strain under pressure to SAW frequency shifts, it reached ~0.2 ppm/KPa for the (0°, 138.5°, 26.7°) cut (Figure 2d). Thus, comprehensively considering the above four factors—*(1) Keff > 0.04; (2) PFA < 1°; (3) low TCF (−10–10 ppm/°C); (4) pressure sensitivity > 0.1 ppm/KPa*—the (0°, 138.5°, 26.7°) cut was confirmed as the optimal choice for the LGS substrate.

### 2.2. Packaging Structure Design and FEM Simulation

**Figure 3:**
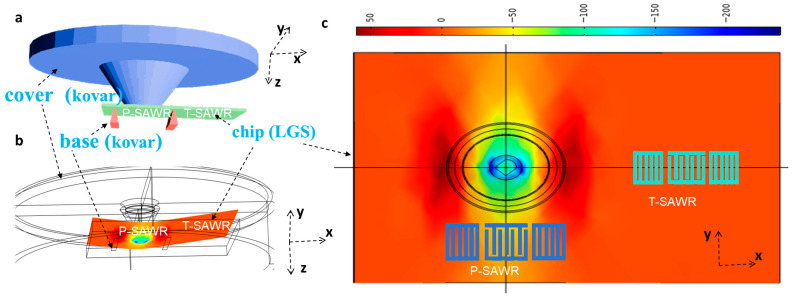
(**a**) Kovar alloy packaging structure integrated with point-force; (**b**) simulated deformation of SAW chip; (**c**) position of two SAW resonators (P-SAWR and T-SAWR).

Traditional LGS SAW pressure sensors rely on sealed-cavity packaging (e.g., Ref. [20]), which suffers from weak pressure-induced substrate deformation, thus limiting sensitivity, making it insufficient for high-precision industrial monitoring. To address this limitation, we proposed a Kovar alloy packaging structure integrated with point-force application (Figure 3a), where Kovar was selected for its thermal expansion coefficient of 5.3 × 10^−6^/°C—closely matching that of LGS (5.1 × 10^−6^/°C) to avoid thermal stress-induced deformation at elevated temperatures (160–400 °C).

The packaging design comprises three key components: a circular cover (radius: 6 mm) with a central probe (diameter: 1 mm) to concentrate pressure onto the LGS substrate; a base (height: 0.3 mm, length: 6.4 mm, width: 4.4 mm) for securing the LGS chip using ceramic adhesive; and a sealed cavity (area: ~6 mm^2^) to isolate the sensor from external contaminants while enabling controlled pressure transmission. To validate the structure’s ability to enhance sensitivity, we modeled its mechanical response under pressure using the static structural module in COMSOL Multiphysics v5.5 (finite element method, FEM). The mesh was refined to 0.1 mm for the LGS substrate to capture localized strain distributions, and 0.2 mm for the Kovar package to balance computational efficiency and simulation accuracy. In the upper cover, fixed boundary conditions were provided around the entire circular cover. Pressure boundary load conditions were applied on the upper surface of the cover. For the rectangular block representing the LGS material, rolling boundary conditions were applied on the two interface surfaces of the tips. Fixed boundary conditions were added on the corners of the LGS substrate to simulate the adhesive. The material coefficients for Kovar were taken from the software’s material library (UNS S30400).

As shown in Figure 3b, application of 1.0 MPa pressure to the cover resulted in the central probe concentrating force on the LGS substrate. Substituting this strain into perturbation theory [24] to calculate pressure sensitivity, the simulated value for the proposed packaging reached 0.196 MHz/MPa (~3.8 ppm/psia)—representing a 270% improvement over conventional sealed-cavity structures (~1.4 ppm/psia) [11] and directly addressing the sensitivity bottleneck of existing designs.

As shown in Figure 3c, two SAW resonators were designed to measure the temperature and pressure simultaneously. One SAW resonator is located in the left part of the chip, where there exists deformation due to the pressure force. This resonator is referred to as P-SAWR. Another SAW resonator is located in the right part of the chip, where there is almost no deformation due to the pressure force because it is fixed to the package base. This resonator is referred to as T-SAWR. Therefore, T-SAWR is almost exclusively sensitive to temperature and can be used to measure temperature. Since the two resonators have the same substrate cut, their frequency–temperature responses are nearly identical. Consequently, the frequency difference of two resonators is almost solely sensitive to pressure and can be used to measure pressure.

### 2.3. SAW Sensor Design

**Figure 4:**
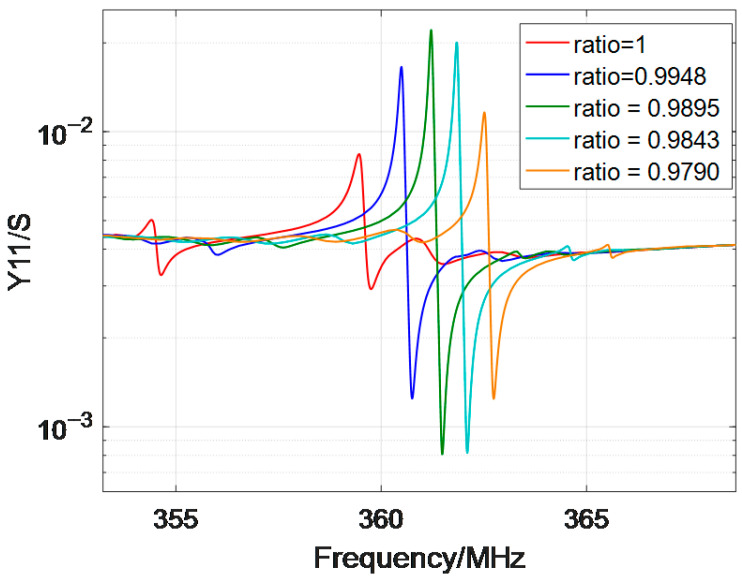
Simulated frequency response of the SAW resonators with different ratios of grating periods to IDT periods.

The SAW resonator was designed using the finite element method/boundary element method (FEM/BEM) to optimize acoustic wave propagation and ensure resonant frequency stability—critical for accurate pressure measurement. The resonator architecture includes two interdigital transducers (IDTs) and two reflective gratings (Figure 3c), with key parameters determined via parametric simulations focused on minimizing spurious modes and maximizing energy confinement (Figure 4).

A critical design parameter was the ratio of grating period to IDT period, as this directly influences stopband insertion loss and resonant purity. Parametric simulations of this ratio with a range from 0.98 to 1.02 were performed using FEM/BEM. A ratio of 0.9895 was found to minimize stopband insertion loss, effectively suppress spurious signals, and ensure pure resonant frequency output. To demonstrate the influence of this ratio on insertion loss, five typical ratio values (0.9790, 0.9843, 0.9895, 0.9948, and 1.0000) were selected, and their calculated admittance modulus (Y11) is shown in Figure 4. Based on this optimal ratio, the IDT was designed with a wavelength of 7.600 μm (to target a center frequency of ~357 MHz), 30 periods (balancing signal amplitude and chip size constraints), and an aperture of 240 μm (reducing edge effects that degrade wave uniformity). Corresponding grating parameters included a wavelength of 7.5202 μm (matching the 0.9895 period ratio), 180 periods (to enhance acoustic reflection efficiency), and a 0 μm gap between the grating and IDT (maximizing acoustic energy confinement within the resonator). The final design parameters of the resonator are summarized in Table 1, which guided subsequent fabrication processes.

## 3. Experimental Procedure

To validate the performance of the proposed LGS SAW pressure sensor, a comprehensive experimental setup was established, encompassing sensor fabrication, packaging assembly, and environmental testing under controlled temperature and pressure conditions. Key processes and equipment specifications are detailed below, with critical configurations aligned to the theoretical design parameters outlined in Section 2.

### 3.1. Sensor Fabrication and Packaging Assembly

The SAW resonator was fabricated on a (0°, 138.5°, 26.7°) LGS substrate (75 mm × 75 mm × 0.5 mm) via a standard lift-off process, consistent with high-temperature SAW device manufacturing protocols. First, the substrate was ultrasonically cleaned in acetone, isopropyl alcohol, and deionized water (10 min each) to remove surface contaminants, ensuring uniform electrode deposition. A 300 nm thick aluminum film was then deposited using magnetron sputtering (SKY Technology Development Co., JGP-560C, Shenyang, China) at 150 W power and 0.5 Pa argon pressure—parameters selected to enhance film adhesion and electrical conductivity. A positive photoresist (MicroChemicals GmbH, AZ 5214E, Westborough, MA, USA) was spin-coated (3000 rpm for 30 s, pre-baked at 90 °C for 1 min), patterned via ultraviolet lithography (MA6, SUSS MicroTec, Garching bei München, Germany) using a design matching Table 1’s resonator parameters, developed (AZ 300 MIF, Darmstadt, Germany, 60 s), and etched with a phosphoric–nitric–acetic acid mixture (8:1:1 volume ratio, 40 °C for 3 min) to define IDTs and gratings. After photoresist stripping (AZ 100 Remover, Darmstadt, Germany, 60 °C for 10 min), the chip was annealed at 200 °C for 2 h in nitrogen to reduce the electrode’s residual stress.

Packaging assembly with the Kovar alloy structure (Section 2.2) followed next. The LGS chip was bonded to the Kovar base using high-temperature ceramic adhesive (H70E, Shanghai Hanxiang, Shanghai, China) cured at 150 °C for 2 h—this adhesive maintains stability up to 500 °C, matching the target 160–400 °C range. Two gold-plated Kovar pins (0.5 mm diameter) were integrated via glass frit technology (450 °C for 30 min) for electrical connections, and aluminum wire bonding (KS8020, Kulicke & Soffa, Santa Ana, CA, USA; 25 μm diameter, bond strength > 5 g per MIL-STD-883H) linked the chip’s electrode pads to the pins. The Kovar cover (with central probe) was laser-welded (YAG laser, Wuhan, China, 1064 nm, 50 W) to the base, forming a hermetic seal with a leak rate < 1 × 10^−9^ Pa·m^3^/s (helium mass spectrometry tested) to protect against external gas interference. Finally, an SMA-connected RF coaxial cable was soldered to the pins for signal transmission.

### 3.2. Experimental Setup and Measurement

**Figure 5:**
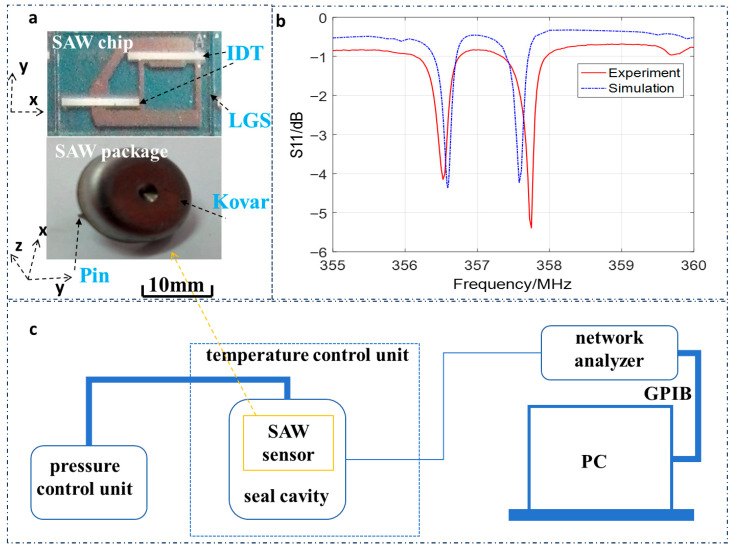
(**a**) The SAW chip and package; (**b**) the simulated and experimental result of the SAW sensor; (**c**) experimental setup for SAW sensor testing.

The characterization system (Figure 5c) included a temperature control unit, pressure control unit, signal measurement module, and data acquisition PC. The sensor was placed in a high-temperature furnace (Carbolite Gero CWF 1300, Sheffield, UK; ±1 °C accuracy) to simulate 160–400 °C conditions (40 °C steps, 30 min holding time per step for thermal equilibrium). A pressure control unit (DPI 612 pump, Druck, Leicester, UK; sealed stainless-steel cavity) regulated pressure from 0 to 1.0 MPa (0.001 MPa resolution, 5 min holding time per point for mechanical stabilization). Sensor signals were transmitted via the RF cable to a vector network analyzer (Keysight E4351C, Santa Rosa, CA, USA, 10 MHz–3 GHz), calibrated with a SOLT kit to eliminate cable/connector errors, which measured S_11_ parameters to capture resonant frequencies. A PC (GPIB interface) automated data collection, with each temperature–pressure combination repeated three times for repeatability.

Data analysis was conducted in MATLAB R2020a: resonant frequencies were extracted via peak fitting to identify the minimum S_11_ value. For temperature response, a third-order polynomial fit modeled the frequency–temperature relationship to quantify and compensate for temperature-induced drift. For pressure response, a linear fit determined sensitivity (slope of the frequency–pressure curve). Discrepancies between experimental and simulated results were attributed to parasitic electromagnetic effects from wire bonding and electrode dimension deviations.

## 4. Results and Discussion

This section presents and analyzes the key performance metrics of the proposed LGS SAW pressure sensor, including resonator quality factor (Q-value), frequency–temperature response, pressure sensitivity, and cross-sensitivity mitigation—validating the effectiveness of the design innovations (Kovar alloy point-force packaging and (0°, 138.5°, 26.7°) LGS cut) and comparing experimental results with theoretical simulations.

### 4.1. Resonator Performance

The SAW resonator’s Q-value and center frequency were first characterized via S_11_ parameter measurements at room temperature (25 °C) and atmospheric pressure. As shown in Figure 5b, the resonator exhibited a sharp resonant dip in the S_11_ curve, with a measured center frequency of ~357 MHz—consistent with the FEM/BEM-simulated value (357.2 MHz) and design parameters in Table 1. The slight deviation was attributed to minor variations in aluminum electrode thickness and LGS substrate elastic constants, which were within an acceptable tolerance for high-frequency SAW devices [17].

The Q-value was calculated as the ratio of the center frequency to the 3 dB bandwidth of the resonant peak, yielding a value of ~3000. This high Q-value indicates low acoustic energy loss, primarily due to two design factors: the (0°, 138.5°, 26.7°) LGS cut’s low acoustic attenuation at high frequencies [17] and the reflective gratings’ optimized period ratio (0.9895), which minimized spurious mode excitation (Figure 4) and confined acoustic energy within the resonator.

### 4.2. Frequency–Temperature Cross-Sensitivity Mitigation

**Figure 6:**
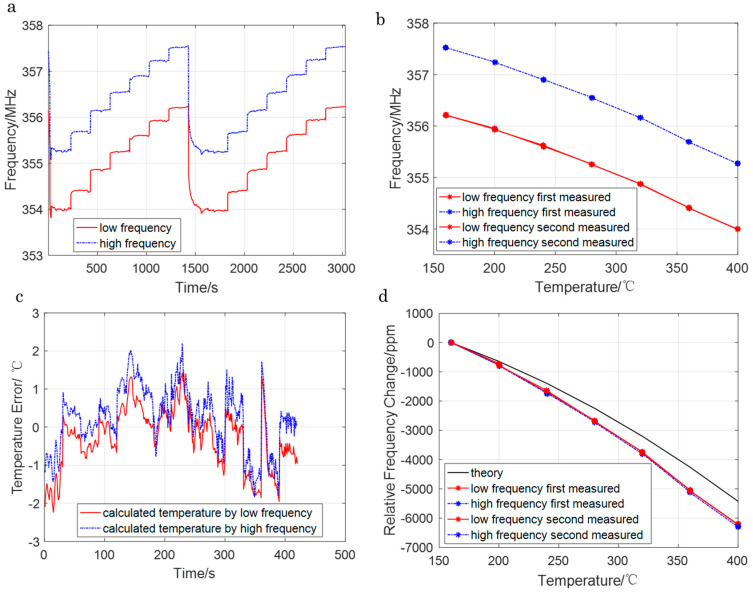
(**a**) Measured frequency response during testing; (**b**) the curve of measured frequency–temperature response; (**c**) the calculated temperature error; (**d**) comparing the experiment and simulation.

To evaluate the sensor’s performance in high-temperature environments, frequency–temperature tests were conducted over 160–400 °C (40 °C intervals) at a constant pressure of 0 MPa, with results shown in Figure 6. The resonant frequency exhibited a monotonic decrease with increasing temperature, consistent with the (0°, 138.5°, 26.7°) cut’s simulated TCF behavior (Figure 2c). The TCF behaviors of two resonators were almost the same, which was compensated for in the pressure calculations. This compensation ensured that pressure-induced frequency shifts were not obscured by temperature effects, confirming the cut’s suitability for high-temperature pressure monitoring.

### 4.3. Pressure Sensitivity and Correlation with Simulations

**Figure 7:**
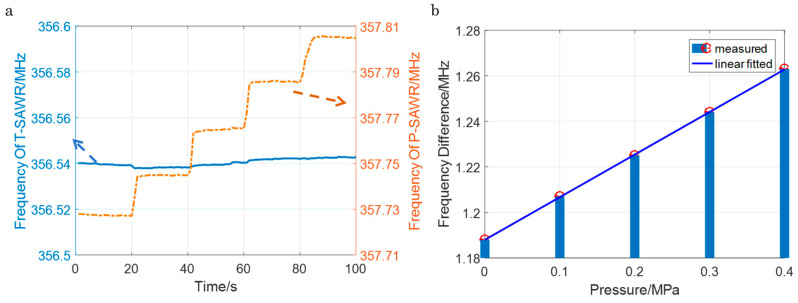
(**a**) The measured frequency response; (**b**) the frequency–pressure curve.

Pressure sensitivity tests were performed over 0–0.4 MPa at a constant temperature of 250 °C (a typical operating temperature for turbine engines and high-temperature manufacturing), with the frequency–pressure curve shown in Figure 7. The resonant frequency increased linearly with pressure, attributed to the Kovar packaging’s point-force design: as pressure increased, the central probe amplified LGS substrate deformation (Figure 3b), altering the substrate’s SAW propagation velocity and thus the frequency [24]. A linear fit to the experimental data yielded a pressure sensitivity of 0.1866 MHz/MPa (R^2^ = 99%), with a standard deviation of ±1%.

This experimental sensitivity was in close agreement with the FEM-simulated value of 0.196 MHz/MPa, with a relative error of only 4.8%. The minor discrepancy was attributed to three factors: (1) parasitic electromagnetic effects from aluminum wire bonds, which introduced small signal losses not accounted for in mechanical simulations; (2) slight deviations in the Kovar probe’s diameter (measured via optical microscopy to be 1.0 ± 0.02 mm) from the simulated 1.0 mm, reducing deformation amplification; and (3) inaccuracies in LGS’s piezoelectric constant used in simulations. Despite these factors, the experimental sensitivity (0.1866 MHz/MPa equivalent to 3.78 ppm/psia) represented a 270% improvement over traditional sealed-cavity LGS SAW sensors (~1.4 ppm/psia [11]), validating the point-force packaging’s ability to enhance the pressure response.

The work described above, conducted at 0–0.4 MPa and 250 °C, validates the high pressure sensitivity of the sensor. However, its measured pressure and temperature ranges are not yet sufficient for the intended application. Future work will focus on optimizing the Kovar packaging’s seal design to extend the pressure-measurement range to 2 MPa and the temperature measurement range above 400 °C, enabling application in high-pressure harsh environments such as rocket combustion chambers. Additionally, integrating a wireless antenna into the packaging will eliminate the need for RF coaxial cables, enhancing the sensor’s suitability for remote monitoring scenarios.

## 5. Conclusions

This study developed and validated a novel langasite (LGS) surface acoustic wave (SAW) pressure sensor optimized for high-temperature harsh environments, addressing key limitations of conventional designs—insufficient pressure sensitivity and pressure-temperature cross-sensitivity—via targeted innovations in substrate selection and packaging structure. The sensor’s core design strengths include the adoption of an LGS substrate with the (0°, 138.5°, 26.7°) cut, which was selected via electromechanical simulations for its superior intrinsic pressure sensitivity and monotonic frequency–temperature response. This cut not only ensured stable acoustic wave propagation but also effectively mitigated temperature-induced frequency drift, a primary source of cross-sensitivity in high-temperature pressure monitoring. Moreover, a Kovar alloy point-force packaging structure was integrated to amplify pressure-induced substrate deformation, directly overcoming the sensitivity bottleneck of traditional sealed-cavity designs.

Comprehensive experimental characterizations confirmed the sensor’s high performance across the target operating range. The SAW resonator achieved a high quality factor of ~3000 at a center frequency of ~357 MHz, indicating low acoustic energy loss and reliable signal output. The sensor exhibited a pressure sensitivity of 0.1866 MHz/MPa, representing a 270% improvement over prior LGS SAW pressure sensors. Additionally, temperature-induced frequency drift was quantified and compensated effectively, resulting in very slight temperature measurement error and further ensuring pressure-measurement accuracy under elevated temperatures.

These results demonstrate that the proposed sensor provides a robust technical solution for accurate, stable pressure monitoring in high-temperature industrial environments, such as turbine engine combustion chambers and high-temperature manufacturing lines. Future work will focus on enhancing long-term durability with the goal of expanding the sensor’s applicability to more extreme harsh-environment scenarios.

## Figures and Tables

**Table 1:** Design parameters of the SAW resonators.

Parameters	P-SAWR	T-SAWR
Center frequency (MHz)	357.6	356.6
Wavelength of IDT (μm)	7.600	7.622
Number of periods of IDT	30	30
Wavelength of grating (μm)	7.5202	7.5420
Number of periods of grating	180	180
Gap between grating and IDT (μm)	0	0
Aperture (μm)	240	240

## Data Availability

The original contributions presented in this study are included in the article. Further inquiries can be directed to the corresponding author.
